# Biodegradation of polychlorinated biphenyls (PCBs) by the novel identified cyanobacterium *Anabaena* PD-1

**DOI:** 10.1371/journal.pone.0131450

**Published:** 2015-07-15

**Authors:** Hangjun Zhang, Xiaojun Jiang, Liping Lu, Wenfeng Xiao

**Affiliations:** 1 College of Life and Environmental Sciences, Hangzhou Normal University, Xuelin Road 16#, Xiasha Gaojiao Dongqu, Hangzhou 310036, Zhejiang Province, China; 2 Key Laboratory of Hangzhou City for Ecosystem Protection and Restoration, Hangzhou Normal University, Hangzhou 310036, China; The University of Iowa, UNITED STATES

## Abstract

Polychlorinated biphenyls (PCBs), a class of hazardous pollutants, are difficult to dissipate in the natural environment. In this study, a cyanobacterial strain *Anabaena* PD-1 showed good resistance against PCB congeners. Compared to a control group, chlorophyll *a* content decreased 3.7% and 11.7% when *Anabaena* PD-1 was exposed to 2 and 5 mg/L PCBs for 7 d. This cyanobacterial strain was capable of decomposing PCB congeners which was conclusively proved by determination of chloride ion concentrations in chlorine-free medium. After 7 d, the chloride ion concentrations in PCB-treated groups (1, 2, 5 mg/L) were 3.55, 3.05, and 2.25 mg/L, respectively. The genetic information of strain PD-1 was obtained through 16S rRNA sequencing analysis. The GenBank accession number of 16S rRNA of *Anabaena* PD-1 was KF201693.1. Phylogenetic tree analysis clearly indicated that *Anabaena* PD-1 belonged to the genus *Anabaena*. The degradation half-life of Aroclor 1254 by *Anabaena* PD-1 was 11.36 d; the total degradation rate for Aroclor 1254 was 84.4% after 25 d. Less chlorinated PCB congeners were more likely to be degraded by *Anabaena* PD-1 in comparison with highly chlorinated congeners. *Meta*- and *para*-chlorines in trichlorodiphenyls and tetrachlorobiphenyls were more susceptible to dechlorination than *ortho*-chlorines during the PCB-degradation process by *Anabaena* PD-1. Furthermore, *Anabaena* PD-1 can decompose dioxin-like PCBs. The percent biodegradation of 12 dioxin-like PCBs by strain PD-1 ranged from 37.4% to 68.4% after 25 days. Results above demonstrate that *Anabaena* PD-1 is a PCB-degrader with great potential for the in situ bioremediation of PCB-contaminated paddy soils.

## Introduction

Polychlorinated biphenyls (PCBs) are a class of anthropogenic chlorinated organic compounds listed as persistent organic pollutants (POP) by the Stockholm Convention on May 22, 2001. These ubiquitous environmental contaminants were dispersed widely in the global ecosystem because of their long half-lives and semi-volatility[1–3]. PCBs are characterized by high lipophilicity, chronic toxicity, and highly stable chemical properties. The high toxicity and bioaccumulation of PCBs throughout the food chain impose a hazardous threat to the biota and the ecosystem [4,5]. Soils are a very important reservoir for PCBs, because these xenobiotics are persistent in soil[6]. A survey of PCB concentrations in 191 surface soil samples revealed that no less than 21,000 tons of PCBs existed in the surface soil globally[7]. What’s more, PCBs can be easily accumulated in soils used to grow rice [8]. A large number of PCB residues have been detected in the paddy fields of south China [9]. In this region, PCB contents in paddy soils were as high as 1636.8 ng/g [10]. A previous study has shown that PCB concentrations in rice were between 41.1 and 132.4 ng/g in the Luqiao and Pingqiao areas of Zhejiang province, where the people’s daily intake of PCBs through rice ingestion reaches up to 12372.9 ng per day [11]. PCBs present a great health risk to the local residents. High concentrations of PCBs can result in neurotoxicity [12], carcinogenesis [13], developmental and reproductive toxicity [14], dermal toxicity, endocrine effects [15], hepatotoxicity, and the induction of diverse phase I and phase II drug-metabolizing enzymes [16]. Increasing public awareness and concern has impelled researchers to identify ways to remove these hazardous organic compounds from the environment.

Many studies on the chemical[17], physical[18], and biological[19] degradation of PCBs have been conducted to remediate these hazardous contaminants. Among these disposal treatments, microbial PCB-degradation is useful and has thus been extensively studied. Harkness et al. [20] found that an indigenous microorganism in the Hudson River can aerobically degrade PCBs at a biodegradation percentage ranging from 37% to 55% after 73 d. Many other microorganisms with the capability of aerobic degradation of PCBs have also been reported, including a strain of *Pseudomonas aeruginosa* [21], *Burkholderia xenovorans* [22], *Arthrobacter* sp. strain B1B, *Ralstonia eutrophus* H850 [23], and *Rhodococcus* sp. strain RHA1 [24]. Most biodegradation research has focused primarily on the use of bacteria and fungi [25,26]. Each isolate shows different spectra in regard to the type and extent of PCB congeners metabolized, with some strains having a narrow spectrum and others being capable of metabolizing a broad range of congeners.

Due to their specific survival and growth conditions and the “flooding-drought” cultivation method in paddy soil, PCB-degraders listed above cannot adapt to the environmental conditions in paddy soil. Thus, they are limited in their application to in-situ bioremediation of PCB-contaminated paddy soils. Fortunately, as ubiquitous groups of organism in the natural environment, microalgae and cyanobacteria have shown great potential for bioremediation applications and are considered capable of degrading chlorinated organic pollutants. Microalga *Scenedesmus obliquus* has the capacity to decompose dichlorophenols[27,28]. Green alga *Chlorella fusca* var. *vacuolata* can degrade dichlorophenol as well[29]. Cyanobacterium *Synechocystis* sp. strain PUPCCC 64 could remove pesticide chlorpyrifos[30]. Two strains of the genus *Anabaena* degraded more than 90% of the organochlorine insecticide endosulfan after 8 d[31]. Cyanobacteria strains also show biodegradability for the organochlorine pesticide lindane [32]. In an investigation of PCB residues in the Taizhou area in Zhejiang, we found that several cyanobacteria strains can grow in PCB-polluted paddy soils. Considering that cyanobacterium has viability when exposed to PCB residues in paddy soils, we proposed the hypothesis that cyanobacteria can dissipate PCBs. To the best of our knowledge, no studies on the degradation of PCBs by cyanobacteria have been reported.

The main objective of this study was to investigate the degradation capability of PCBs by *Anabaena* PD-1 isolated from paddy soil in liquid cultures. Chlorine-free culture medium was used to study the tolerance of strain *Anabaena* PD-1 against PCBs. Sequence analysis of 16S rRNA was utilized to identify this PCB-degrading cyanobacterium. A study of Aroclor 1254 and dioxin-like PCBs degradation by cyanobacterium strain PD-1 was also conducted through solid-phase extraction and gas chromatography. This study could provide a new knowledge for PCB-biodegradation and establish the application of dechlorination functional cyanobacteria for biological disposal of persistent organic wastes.

## Material and Methods

### Chemicals

Aroclor 1254 was purchased from Sigma-Aldrich (St. Louis, MO, USA). The dioxin-like PCBs mixture standards including PCB77, PCB81, PCB105, PCB114, PCB118, PCB123, PCB126, PCB156, PCB157, PCB167, PCB169, and PCB189 were purchased from Aladdin Reagent (Shanghai, China). The purities of all PCB standards were >99%. The International Union of Pure and Applied Chemistry (IUPAC) numbers are used to identify the PCB congeners. Methanol and *n*-hexane purchased from Huadong Pharmaceutical (Hangzhou, China) were of GC- or HPLC-grade. All the other reagents were of analytical grade.

### Cyanobacterial strain and culture conditions

*Anabaena* PD-1 was isolated from PCB-contaminated paddy soils in Taizhou, Zhejiang, China (No specific permissions were required for the sampling locations and activities. The field studies did not involve endangered and protected species. The sampling site in the study is located at Latitude 28°32’N Longitude 121°27’E.). *Anabaena* PD-1 cells were grown at 25°C, 2000 lux, in BG11 liquid enrichment media[33]. One liter of the BG11 medium contained 0.04 g K_2_HPO_4_, 0.075 g MgSO_4_·7H_2_O,0.036 g CaCl_2_·2H_2_O, 6.0 mg citric acid, 6.0 mg ferric ammonium citrate, 1.0 mg Na_2_EDTA, 0.02 g Na_2_CO_3_, and 1.0 mL trace element solution A_5_. One liter of the trace element solution A_5_ contained 2.86 g H_3_BO_3_, 1.81 g MnCl_2_·4H_2_O, 0.222 g ZnSO_4_·7H_2_O, 0.39 g Na_2_MoO_4_·2H_2_O, 0.079 g CuSO_4_·5H_2_O, and 49.4 mg Co(NO_3_)_2_·6H_2_O. Culture temperature and illumination intensity were set to 25±2°C and 2000 lux, respectively. The light to dark ratio was 12h: 12h.

All the *Anabaena* PD-1cyanobacteria cells were cultured with the chlorine-free medium. The cultured cyanobacteria cells were divided into four groups (I, II, III and IV), and each group was treated with different concentrations of Aroclor 1254. There were another three Aroclor 1254 control groups (CII, CIII and CIV) without any cyanobacteria cells in the chlorine-free mediums. Groups and treatments were set up as shown in Table 1.

**Table 1:** Treatment protocols of *Anabaena* PD-1 cells and Aroclor 1254 in different groups.

Treatment	Group I	Group II	Group III	Group IV	Group CII	Group CIII	Group CIV
Aroclor 1254 (mg/L)	0	1	2	5	1	2	5
PD-1 cells	+	+	+	+	-	-	-

‘+’ and ‘-’ mean the group was cultured with or without PD-1 cells.

### Determination of chlorophyll a content

Cyanobacterial cells in the exponential growth phase (OD_680_ = 0.38) were exposed to 1, 2, and 5 mg/L Aroclor 1254 for 7 days. Chlorophyll *a* was determined using a modified hot-ethanol extraction method[34]. To determine chlorophyll *a* content, the cyanobacterial cultures were harvested and disrupted by ultrasonication for 30 min. After that, the chlorophyll *a* was extracted using 15 mL hot ethanol (70% v/v, 70°C) in a preheated water bath for 15 min and then in dark place for 6 h. The absorption of extracts was measured at 665 nm and 750 nm by an ultraviolet spectrometry (Shimazu Co. Japan). Four independent extraction and adsorption measurement experiments were performed for each group.

### Dechlorination of PCBs by *Anabaena* PD-1

A chlorine-free medium was utilized to detect the PCB degradation ability of *Anabaena* PD-1. The chlorine-free medium was modified BG11 medium without addition of CaCl_2_·2H_2_O and trace element solution. One liter of the chlorine-free BG11 medium contained 0.04 g K_2_HPO_4_, 0.075 g MgSO_4_·7H_2_O, 0.040 g Ca(NO_3_)_2_, 6.0 mg citric acid, 6.0 mg ferric ammonium citrate, 1.0 mg Na_2_EDTA, 0.02 g Na_2_CO_3_, and 1.0 mL trace element solution A_5_. One liter of the trace element solution A_5_ contained 2.86 g H_3_BO_3_, 1.64 g Mn(NO_3_)_2_, 0.222 g ZnSO_4_·7H_2_O, 0.39 g Na_2_MoO_4_·2H_2_O, 0.079 g CuSO_4_·5H_2_O, and 49.4 mg Co(NO_3_)_2_·6H_2_O. Otherwise, the culture condition was the same as described above. The transparent conical flasks (100 mL) were used to contain the cultures and the degradation experiments were performed under anaerobic condition.

According to Table 1, Aroclor 1254 was added into 20 mL cultures with or without *Anabaena* PD-1 cells (OD_680_ = 0.38). The concentrations of Aroclor 1254 were adjusted to 1, 2, and 5 mg/L by methanol, respectively. After 7-day exposure, the cyanobacterial cultures in control and PCB-treated groups were centrifuged for 10 min at 4000 rpm. A modified mercuric thiocyanate-ammonium ferric sulfate spectrophotometry method was used to test the concentration of chloride ion in the solution [35]. Five mL supernate was used to measure absorption at 460 nm by an ultraviolet spectrometry (Shimazu Co. Japan). Four independent experiments were performed for each group.

### Identification of PCB-degrading cyanobacterium

Total RNA of strain *Anabaena* PD-1 was extracted using a general gene extraction kit (Haoji Biotechonlogy, Hangzhou, China). Then the RNA was amplified by PCR using universal 16S rRNA primers (F: 5’-GAGTTTGATCCTGGCTCAG-3’) and (R: 5’-AGAAAGGAGGTGATCCAGCC-3’). The 16S rRNA products were sequenced on an Applied Biosystems (Foster, Calif., USA) automatic sequencer. The thermal-cycling conditions were 94°C for 4 min; 35 cycles; 94°C for 30 s, 57°C for 30 s, 72°C for 2 min. PCR was performed in at least two independent experiments.

The 16S rRNA sequences were compared and aligned with sequences deposited in the GenBank database using the BLAST program. The 16S rRNA of *Anabaena* PD-1 and other related cyanbacterial sequences retrieved from the NCBI database were aligned and analyzed using PAUP 4.0 beta, and these aligned sequences were used to construct a phylogenetic tree using the neighbor-joining [36] and Jukes-cantor distance correction matrix methods. The branching pattern was verified using 1000 bootstrap replicates.

### PCBs extraction and gas chromatography (GC) analysis

To examine the PCB residues in the degradation system, the culture of *Anabaena* PD-1 exposed to Aroclor 1254 in the conical flask were directly extracted with *n*-hexane after ultrasonication for 30 min. The Aroclor 1254 residues in the clear *n*-hexane extracts were separated in a separatory funnel, and the extracts were dried over 5 g anhydrous sodium sulfate and 5 g silica gel. Then the extracts were nitrogen-dried and dissolved in 1 mL *n*-hexane for GC analysis.

A 1 μL aliquot of each extract was injected into an Agilent 6890 series gas chromatograph equipped with q capillary column (HP-5 fused-silica, 30 m × 0.32 mm × 0.25 μm) with an electron capture detector (Agilent Technologies, USA). According to a previous study[37], the amended parameters for GC detection were set as follows: the injection temperature was 300°C, and the detector temperature was 300°C; the column temperature was initially held at 90°C; ramped at 15°C/min to 180°C and then at 1°C/min to 220°C, held for 5 min; and then at 7°C/min to 290°C and held for 2 min. High-purity nitrogen as carrier gas was maintained at a constant flow rate of 1 mL/min. Both 2,4,5,6-tetrachloro-*m*-xylene (TMX) and PCB 209 were used as the internal standards. The recoveries of PCB congeners ranged from 93.3% to 103.5%. The detection limit of this method for all the PCB congeners in Aroclor 1254 was about 1 ng/L.

### Biodegradation kinetics of Aroclor 1254 by *Anabaena* PD-1

Cells of *Anabaena* PD-1 during the exponential phase were applied for the experiment. Four hundred μL Aroclor 1254 (100 mg/L, dissolved in methanol) were added to the cell suspensions. Control cells were inactivated by autoclaving at 120°C for 30 min prior to the addition of Aroclor 1254. Cells were incubated in conical flasks for 0, 1, 2, 5, 10, 15, 20, and 25 d at 25±2°C in a phytotron with a light to dark ratio of 12h: 12h. For each experiment, the initial Aroclor 1254 concentration was adjusted to 2 mg/L.

The biodegradation kinetics of Aroclor 1254 was fitted by the equation *C*_t_ = *C*_0_e^k*t*^, where *C*_0_ and *C*_t_ are the initial and biodegraded concentrations of Aroclor 1254, respectively. The biodegradation half-life (*T*_1/2_) of Aroclor 1254 was calculated using the equation $T_{1/2}=\frac{\ln\;2}{\text{k}}$, where k is the biodegradation reaction coefficient. Percent biodegradation of PCB congeners were calculated according to the mass percentage of individual PCB congeners in the mixture.

### Determination of dioxin-like PCBs

In order to determine the biodegradation effects of 12 kinds of dioxin-like PCBs by *Anabaena* PD-1 cells, cyanobacterial cells in the exponential phase were used. Four hundred μL dioxin-like PCBs (100 mg/L, dissolved in methanol) were added to 20 mL cyanobacterial cultures (OD_680_ = 0.38). Total PCBs concentration was adjusted to 2 mg/L. Control cells were inactivated by autoclaving at 120°C for 30 min prior to the addition of dioxin-like PCBs. After incubation for 25 d, the cyanobacterial cultures was subjected to ultrasound and extracted for GC/MS detection. The extracts were clean-up and applied to a SPE-NH_2_ cartridge (Varian). The filtered extract was then concentrated under nitrogen and refilled with 100 μL of hexane. Four independent experiments were performed for each group.

Quantification of dioxin-like PCBs was accomplished by use of previously established method [38]. GC/MS analyses were performed on an Agilent Technologies 7890 gas-chromatograph coupled with a 5973 mass spectrometer using a DB5MS column (60 m × 0.25 mm ID × 0.25 μm film, Agilent Technologies, Palo Alto, CA, USA)). Details of the chemical analysis (**S1 File**), as well as recoveries can be found in the supporting information (**S1 Table**).

### Statistical analysis

The statistical differences of the experimental data were determined using one-way ANOVA followed by two-sided Dunnett’s t-test. Statistical tests were conducted using SPSS11.0, and the statistical significance values were defined as *P<0.05 and **P<0.01. All data of repeated experiments were presented as mean±standard deviation (S.D.) in quadruplicate.

## Results

### Effect of Aroclor 1254 on cyanobacterial growth

**Fig 1:**
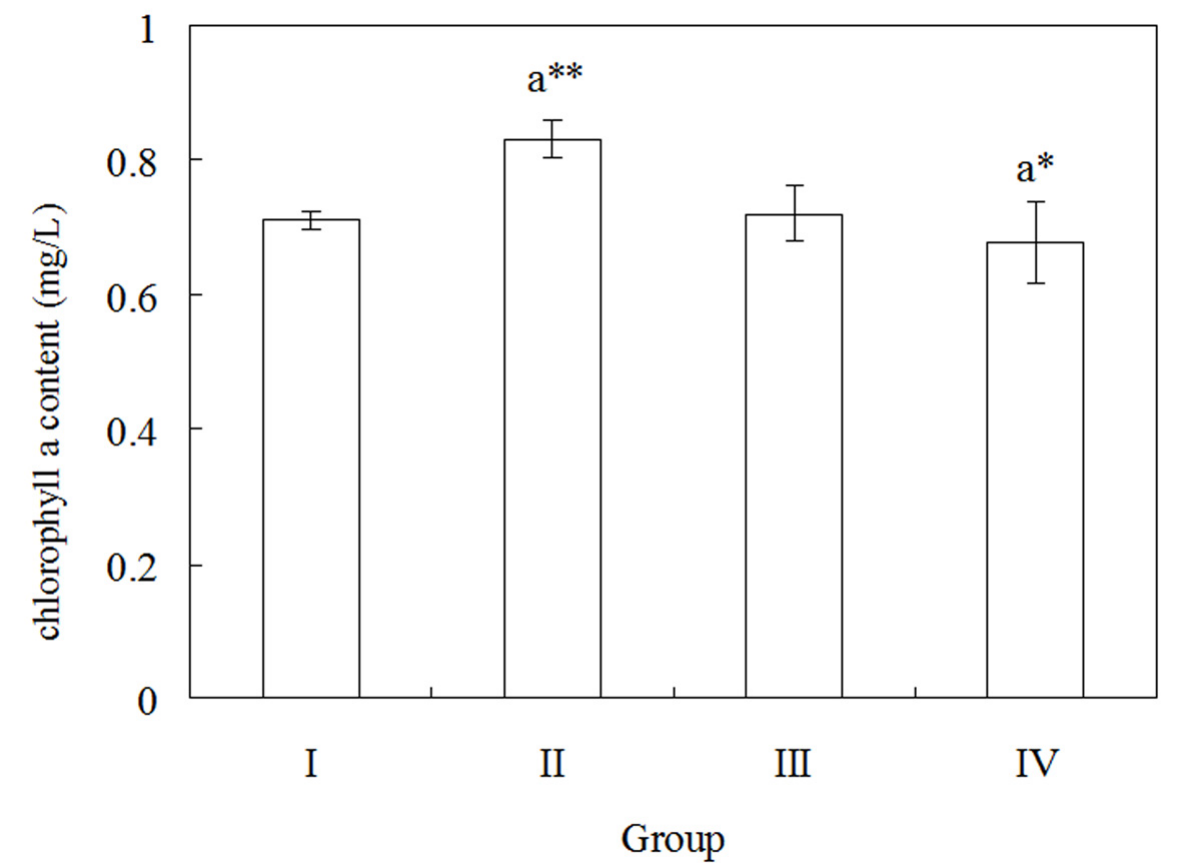
The changes of chlorophyll *a* in cells of cyanobacterium *Anabaena* PD-1 exposed to different concentrations of Aroclor 1254. The result was presented as the mean values of quadruplicate experiments (**P<0.01, *P<0.05; ‘a’ indicates group II, III, IV vs. the control group I).

Chlorophyll *a* is an important index to estimate the biomass of phytoplankton including cyanobacteria[39]. In addition, chlorophyll is a sensitive parameter used to examine microalgae response to toxic organic contaminats. Thus, changes of chlorophyll *a* content could reflect cyanobacterium *Anabaena* PD-1 response to stress induced by PCBs. As shown in Fig 1, the chlorophyll *a* content in the group II increases to 0.82 mg/L. It is significantly higher than that in the group I (*p*<0.01), which indicates that cyanobacterium *Anabaena* PD-1 can stress under low dose exposure of Aroclor 1254. Nevertheless, when cyanobacterium *Anabaena* PD-1 is exposed to 5 mg/L Aroclor 1254 after 7 days, the chlorophyll *a* content is only 0.67 mg/L. It is significantly lower than that in the group I (*p*<0.05), which means higher dose exposure of Aroclor 1254 may affect the growth of cyanobacterium *Anabaena* PD-1. And there is no significant difference of the chlorophyll *a* content between in the group III and in the group I. It suggests that cyanobacterium *Anabaena* PD-1 can tolerate 2 mg/L Aroclor 1254.

### Biodegradation efficiency of Aroclor 1254 by *Anabaena* PD-1

**Fig 2:**
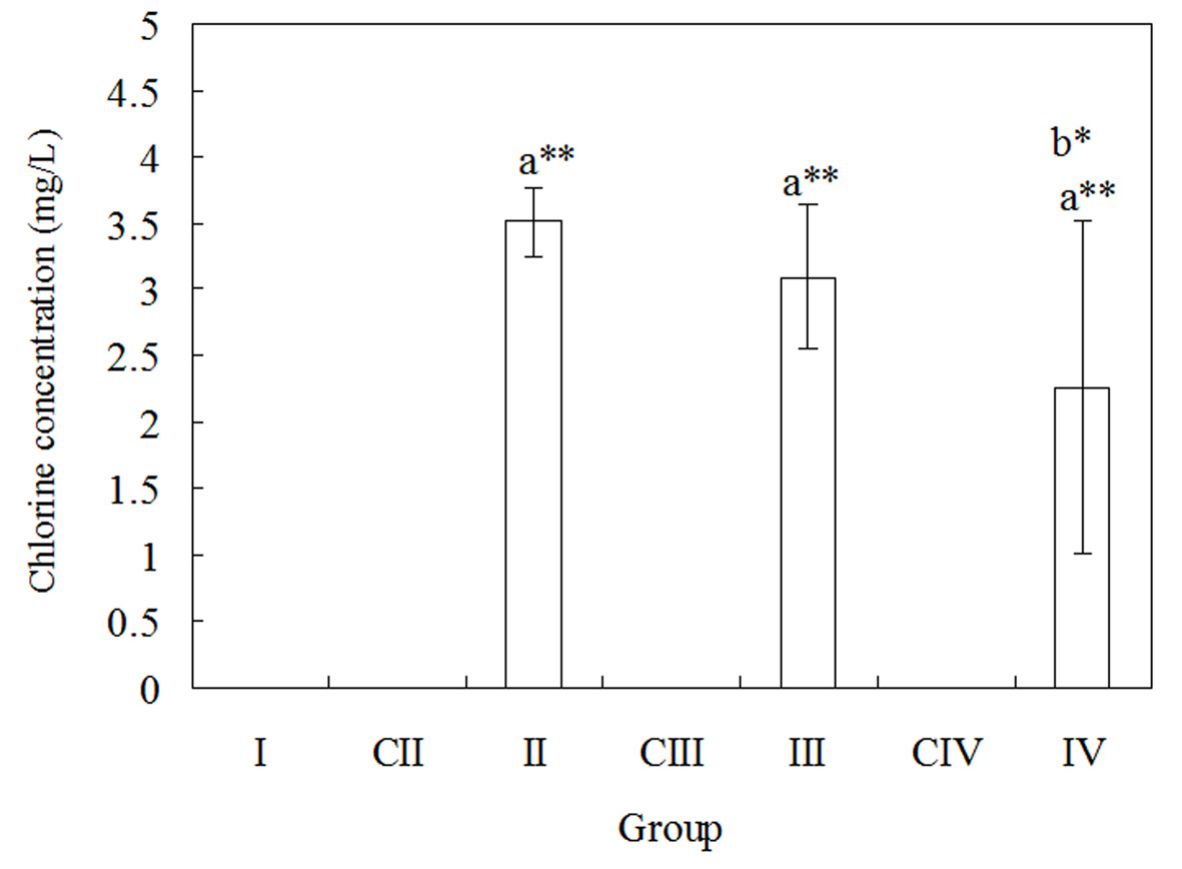
Dechlorination effects of Aroclor 1254 by *Anabaena* PD-1 cells. The result was presented as the mean values of quadruplicate experiments (**P<0.01, *P<0.05; ‘a’ indicates group II, III, IV vs. the control group I; ‘b’ indicates group III, IV vs. group II).

Changes of chloride ion concentration in cyanobacterial chlorine-free cultures exposed to 1, 2, and 5 mg/L Aroclor 1254 is presented in Fig 2. On incubation day 7, the Cl^-^ concentration of the PCB-treated groups increased significantly compared with the control group. When *Anabaena* PD-1 was exposed to 1 mg/L PCB, the maximum Cl^-^ concentration reached 3.55 mg/L. At PCB exposure concentrations of 2 and 5 mg/L, the Cl^-^ concentrations were 3.05 and 2.26 mg/L, respectively. Results above clearly proved that cyanobacterium *Anabaena* PD-1 could degrade PCB congeners via dechlorination.

### 16S rRNA sequence analysis and identification of *Anabaena* PD-1

As shown in Fig 2, it is clearly demonstrated that *Anabaena* PD-1 can degrade PCBs. The PCR products were linked to pUCm-T carrier, and sequencing was performed after purification and recycling. The detailed 16S rRNA sequencing results are in the supporting information (**S2 File**).

**Fig 3:**
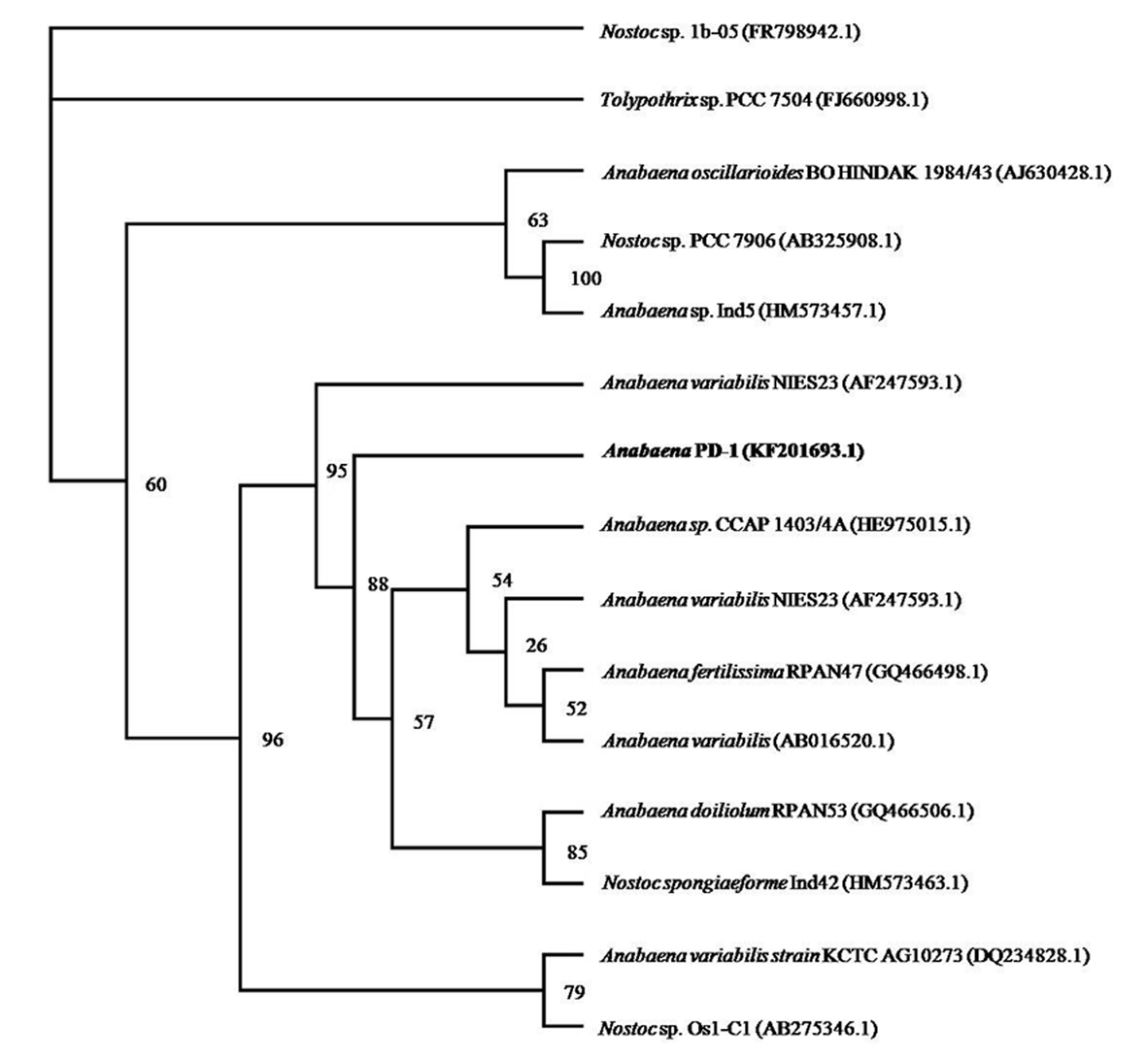
Phylogenetic tree based on an alignment of the sequences of the 16s rDNA of cyanobacterium *Anabaena* PD-1, which was generated using PAUP. The cyanobacterial strain obtained and the accession number in this study are shown in bold.

The 16S rRNA gene sequences of *Anabaena* PD-1 were aligned with the sequences deposited in the GenBank database using a BLAST search. The results of the phylogenetic analysis showed that strain PD-1 belongs to the genus *Anabaena* (Fig 3). The 16S rRNA gene sequence of *Anabaena* PD-1 was submitted to the GenBank. The accession number 16S rRNA of strain PD-1 is KF201693.1. This strain was preserved in the China Center for Type Culture Collection with accession number M 2011446.

### Biodegradation of Aroclor1254

**Fig 4:**
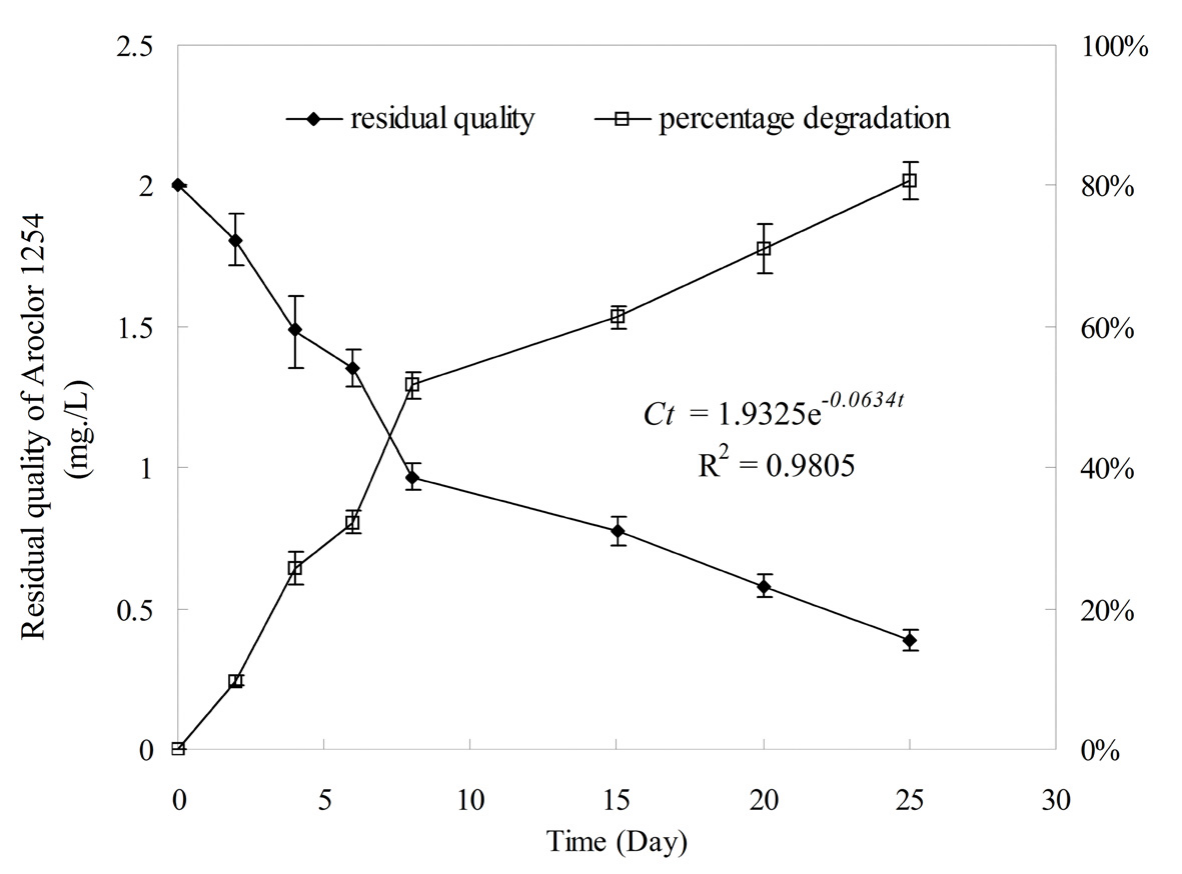
The biodegradation kinetics of Aroclor 1254 by *Anabaena* PD-1. The result was presented as the mean values±standard deviation (S.D.) of quadruplicate experiments.

The biodegradation dynamics of Aroclor 1254 by *Anabaena* PD-1 is presented in Fig 4. After 25 d of incubation, the total concentration of Aroclor 1254 observably decreased. The total Aroclor 1254 removal efficiency of *Anabaena* PD-1 was approximately 85%. The degradation fit the first order reaction kinetics equation described as 1.4898e^-0.0614k*t*^ (*R* ^*2*^ = 0.974). The calculated half-life for the biodegradation was 11.36 d. This strain degradation was detectable in mono-, di-, tri-, tetra-, penta-, and hexachlorobiphenyls for 25 d (Table 2), and almost all the tri- and pentachlorodiphenyl congeners of Aroclor 1254 were extensively degraded (greater than 80%). Within 6 d, *Anabaena* PD-1 degraded the major peaks to a certain degree with 2 mg/L Aroclor 1254.

**Table 2:** Biodegradation percentages of 2 mg/L total PCB congeners with different chlorine substituted positions by *Anabaena* PD-1 for 25 days.

Cl number	IUPAC #	Cl position	Degradation percentage (%)
3	16	23–2	75.0
	17	24–2	100
	18	25–2	100
	19	26–2	61.8
	22	23–4	95.8
	24	236	100
	27	26–3	100
	28	24–4	100
	31	25–4	97.3
	32	26–4	75.0
	33	34–2	100
	35	34–3	95.9
4	40	23–23	98.9
	41	234–2	95.7
	43	235–2	100
	44	23–25	92.9
	45	236–2	68.5
	46	23–26	100
	48	245–2	100
	49	24–25	98.3
	51	24–26	95.8
	52	25–25	100
	56	23–34	100
	60	234–4	100
	61	2345	100
	64	236–4	95.7
	70	25–34	100
	74	245–4	86.6
5	83	235–23	69.7
	85	234–24	62.1
	87	234–25	68.3
	91	236–24	84.5
	95	236–25	90.6
	97	245–23	67.0
	99	245–24	75.2
	101	245–25	78.4
	102	245–26	90.6
	105	234–34	40.2
	110	236–34	30.8
	119	246–34	69.7
	121	246–35	82.9
	122	345–23	53.0
6	128	234–234	17.8
	130	234–235	36.2
	131	2346–23	53.0
	132	234–236	40.2
	134	2356–23	45.4
	137	2345–24	43.4
	138	234–245	31.0
	140	234–246	57.6
	141	2345–25	36.9
	146	235–245	42.6
	149	236–245	38.0
	151	2356–25	76.8
	153	245–245	34.5
	159	2345–35	28.9
7	172	2345–235	25.1
	174	2345–236	24.7
	175	2346–235	36.7
	177	2356–234	25.4
	178	2356–235	35.9
	180	2345–245	28.3
	183	2346–245	19.0
	185	23456–25	27.1

The biodegradation of PCBs with different positions of chlorine substitution in the benzene ring by *Anabaena* PD-1 was examined. PCB congeners with different degradation percentages were used for this analysis. The results are shown in Table 2, which indicates that *Anabaena* PD-1 exhibits greater dechlorination activity toward PCB congeners with *meta*- and *para*-chlorine-substituted congeners than those with *ortho*-chlorine-substituted congeners.

To investigate the influence of PCBs with different chlorine substituent numbers on the degradation by *Anabaena* PD-1, the PCB congeners were separated into five groups (tri-, tetra-, penta-, hexa-, and hepta-PCBs) according to their chlorine substituent numbers (Table 2). As can be seen from Table 2, *Anabaena* PD-1 exhibited greater degrading capability against tri- to pentaCB congeners than PCB congeners substituted with six or more chlorine atoms. After exposure in Aroclor 1254 for 25 d, the percent biodegradation of triCBs, tetraCBs, and pentaCBs ranged from 61.8% to 100%, 66.8% to 100%, and 30.8% to 90.6%, respectively, whereas the maximum degradation percentage of hexaCB (PCB151) is 76.8%. These results clearly demonstrate that for the degradation of PCBs by cyanobacteria like *Anabaena* PD-1 the degradation effects for each individual PCB congeners depend on the chlorine substituent positions in biphenyl rings.

### Degradation of dioxin-like PCBs by *Anabaena* PD-1

**Fig 5:**
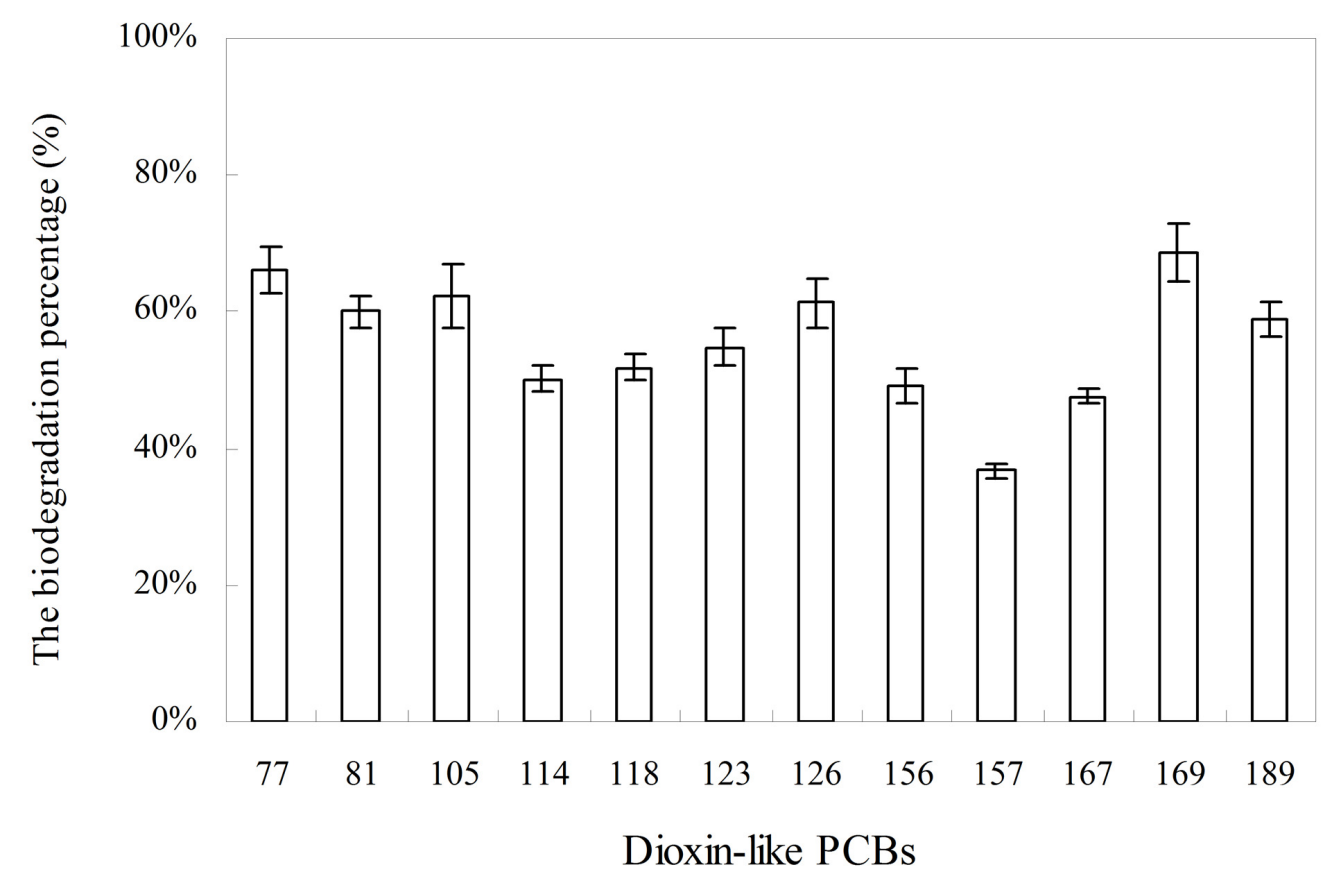
The biodegradation percentage of dioxin-like PCBs degraded by *Anabaena* PD-1 for 7 days. The total initial exposure concentration for dioxin-like PCBs is 2 mg/L. The result was presented as the mean values of quadruplicate experiments.

Fig 5 shows the degradation capability of *Anabaena* PD-1 on dioxin-like PCBs. The degradation percentages of dioxin-like PCBs range from 37.3% to 68.4%, which indicates the favorable PCB detoxification capability of *Anabaena* PD-1. In addition, the cyanobacterial stain showed a slightly higher degradation rate of lower chlorinated dioxin-like PCBs than the highly chlorinated ones. However, the highest rate was exhibited by PCB169, which suggests the possible relationship between degradation rate and chlorine substituent position.

## Discussion

The cyanobacterial strain *Anabaena* PD-1 originally isolated from PCB-contaminated paddy soils is the first cyanobacterium identified that is capable of PCB congeners degradation. As shown in Fig 1, the chlorophyll-a content in the group II is significantly higher than that in the control group (*p*<0.01). It demonstrates that under the exposure of 2 mg/L Aroclor 1254 *Anabaena* PD-1 can tolerate PCBs. The results of chloride ion content in chlorine-free medium (Fig 2) indicate that *Anabaena* PD-1 exhibits PCB dechlorination capability. Nevertheless, the chlorophyll-a content in the group IV is significantly lower than that in the control group (*p*<0.05). It means that when the exposure concentration of Aroclor 1254 is higher than 5 mg/L, PCBs may inhibit the growth of *Anabaena* PD-1. Thus, as shown in Fig 2, the chlorine concentration decreases with increasing PCBs concentration. The chlorine concentration in the group IV is significantly lower than that in the group II (*p*<0.05).

As PCB-degraders, many microorganisms have resistance against PCBs stress[40]. PCBs have been reported to impair the growth and survival of PCB-degrading microbes [41, 42]. Different kinds of bacteria show various levels of tolerance to PCBs. The aerobic PCB-degrading bacteria *B*. *xenovorans* LB400 is highly resistant to the toxicity induced by PCBs and can thus survive when exposed to 500 mg/L Aroclor 1242 [43]. *Pseudomonas* sp. strain B4 shows tolerance to 2 mM 2-chlorobiphenyl[44]. Besides, plant growth-promoting rhizobacteria *Pseudomonas fluorescens* F113[45], *Pseudomonas putida* Flav1-1 and *Pseudomonas putida* PML2[46] have tolerance to PCB stress as well. However, growth of microalgae *Cholrella pyrenoidosa* and *Scenedesmus obliquus* is inhibited by Aroclor 1254 (> 2 mg/L)[47]. In this study, the adverse effect of PCBs on *Anabaena* PD-1 was detected by measuring the contents of chlorophyll *a* (Fig 1). These results imply that *Anabaena* PD-1 can tolerate the toxicity of PCB congeners and even degrade PCBs.

According to the results shown in Table 2, *Anabaena* PD-1 is confirmed as a new strain that can efficiently degrade both low- and high-chlorinated PCBs. It is clearly demonstrated that *Anabaena* PD-1 has stronger PCB commercial mixtures degradation capability than some PCB-degrading bacterial strains and fungi by comparing the degradation substrates and degradation time. According to Boyle et al. [48], *Comamonas testosteroni*, *Rhodococcus rhodochrous*, and a strain of *Pseudomonas putida* can metabolize Aroclor 1242 with total Aroclor 1242 losses of 13.8%, 19.1%, and 24.6%, respectively. Singer and co-workers conducted a study that used *Arthrobacter* sp. strain B1B and *R*. *eutrophus* H850 for Aroclor 1242 degradation[49]. The biodegradation percentages of the PCB commercial mixture by these strains is from 55% to 59%, lower than the decomposition percentages of Aroclor 1254 by *Anabaena* PD-1. In addition, Yadav et al. [50] found that 30.5% of Aroclor 1254 was degraded by the white rot fungus *Phanerochaete chrysosporium*. The results in Fig 4 show that strain PD-1 has a stronger capability for Aroclor 1254 degradation than *P*. *chrysosporium*. Thus, besides the aforementioned bacterial strains and fungi, the cyanobacterium *Anabaena* PD-1 may be a novel tool to the biodegradation of PCBs.

The results in Table 2 also present the biodegradation percentages of different kinds of PCB congeners. These results show that the strain PD-1 can degrade an extremely wide range of congeners in Aroclor 1254. Strain PD-1 not only can degrade low-chlorinated products but also can decompose the highly chlorinated congeners, including hexa- and hepta- chlorobiphenyls. The degradation percentages of tri-, tetra-, and penta- chlorobiphenyls range from 99.3% to 72.1%. Strain PD-1 shows strong capability to degrade PCB congeners with five or fewer chlorines. The biodegradation effects of PCB congeners are depended on different PCB-degraders and the different experimental conditions. *Ralstonia* sp. SA-3 isolated from PCB-contaminated African system cannot degrade PCB91 and PCB99 in Aroclor 1242[51]. However, the biodegradation percentages of PCB91 and PCB99 by *Anabaena* PD-1 are 84.5% and 75.2%, respectively. In addition, the 20-day biodegradation percentage of Aroclor 1254 by *Ceriporia* sp. ZLY-2010 is only 7.65%[52], while 84.4% of total Aroclor 1254 is transformed by *Anabaena* PD-1 after 25 d. Therefore, *Anabaena* PD-1 exhibit its advantage on different type of PCB congeners. On some tri-, tetra-, and pentachlorobiphenyls, such as 2,2’,6-trichlorobiphenyl, 2,3,6-trichlorobiphenyl, 2,2’,3,3’-tetrachlorobiphenyl, and 2,2’,4,5,5’-pentachlorobiphenyl, *Anabaena* PD-1 shows biodegradation activities superior or equivalent to those of the previously reported strong PCB degraders, including *Acinetobacter* sp. P6 and *Alcaligenes eutrophus* H850 (Table 3). Moreover, compared with *Acinetobacter* sp. P6 and *A*. *eutrophus* H850, *Anabaena* PD-1 exhibits higher broad-spectrum degradation capability for Aroclor 1254.

**Table 3:** Comparison degradation effects of PCBs degraded by *Anabaena* PD-1 and other microorganisms.

IUPAC #	Congener assignment(s)	Cl No.	*Anabaena* PD-1 ^a^	P6^b^	H850^c^
PCB-31	25–4	3	61	-	100
PCB-28	24–4	3	100	-	45
PCB-33	34–2	3	100		100
PCB-52	25–25	4	52	77	100
PCB-49	24–25	4	49	49	95
PCB-44	23–25	4	51	100	100
PCB-41	234–2	4	51	100	100
PCB-64	236–4	4	47	21	15
PCB-40	23–23	4	100	88	-
PCB-74	245–4	4	39	99	-
PCB-70	25–34	4	37	100	95
PCB-102	245–26	5	40	63	50
PCB-95	236–25	5	40	63	50
PCB-91	236–24	5	39	12	-
PCB-60	234–4	4	39	12	-
PCB-56	23–34	4	33	80	20
PCB-99	245–24	5	35	-	20
PCB-119	246–34	5	100	-	20
PCB-97	245–23	5	60	99	25
PCB-87	234–25	5	30	-	60
PCB-136	236–236	6	37	-	20

a: Data from this study; *Anabaena* PD-1 was exposed to 2 mg/L Aroclor 1254 for 6 days.

b: Data from Kohler et al., [53]; P6 was exposed to 10 mg/L Aroclor 1254 for 6 days.

c: Data from Bedard et al., [54]; H850 was exposed to 10 mg/L Aroclor 1254 for 3 days.

The degradation pattern of PCB congeners with different chlorines by *Anabaena* PD-1 shows that the degradation rate decreases as the number of chlorine atoms in the biphenyl ring increases, which is consistent with other studies [55].The chlorine number on the biphenyl ring can generally influence the PCB dechlorination activity of *Anabaena* PD-1. Meanwhile, the arrangement of chlorines on the phenyl ring can also affect dechlorination. Table 2 presents the biodegradation patterns for PCB congeners with different chlorine substituted positions. A previous study has shown that PCB congeners with two *ortho* chlorines (2, 2’ or 2, 6) are rarely degraded by most bacterial strains that can degrade PCBs [56]. A high degree of chlorination has been proven to require more energy to break the stable carbon-chlorine bonds [57]. Similarly, our results show that trichlorodiphenyls and tetrachlorobiphenyls with fewer *ortho*-chlorines are more susceptible to degradation. For example, PCB19, which has three *ortho*-chlorines, has a degradation rate (61.8%) lower than that of trichlorodiphenyls with two or one *ortho*-chlorines such as PCB17 (100%), PCB18 (100%), and PCB28 (100%). The biodegradation rates of PCB41 and PCB60 are 95.7% and 100%, respectively, with no significant difference. Compared with PCB45, with total loss of 68.5% suggests that *Anabaena* PD-1 has prior *meta*- and *para*-chlorine dechlorination ability than *ortho*-chlorines. This dechlorination pattern is supported by Wiegel, who found that dechlorination activity generally occurred in *meta*- and *para*-chlorines of the biphenyl molecule [58]. In addition, the dechlorination of hexachlorobiphenyls and heptachlorobiphenyls by *Anabaena* PD-1 shows no significant pattern.

As a strong PCB-degrader, *Anabaena* PD-1 shows detoxification capability for dioxin-like PCBs as well. Dioxin-like PCBs are widespread in a global scale [59], and the residual dioxin-like PCBs in the environment is generally higher than that of the dioxins [60]. Given this high toxicity, most bacterial strains that can efficiently degrade many other PCB congeners cannot decompose dioxin-like PCBs effectively. In this study, the PCB-degrading algae *Anabaena* PD-1 not only can tolerate dioxin-like PCBs but can also degrade these highly hazardous pollutants. PD-1 can degrade all 12 kinds of dioxin-like PCBs. The biodegradation rate of PCB169 is 68% on incubation day 7. To a certain extent, *Anabaena* PD-1 can detoxicate dioxin-like PCBs and then reduce the risks of dioxin-like PCBs to the environment and ecology.

## Conclusions

In this study, the novel cyanobacterium *Anabaena* PD-1 isolated from contaminated paddy soils was identified as a strain of the genus of *Anabaena* using morphology and 16S rRNA sequencing analysis. *Anabaena* PD-1 is capable of degrading PCBs. The biodegradation rate of Aroclor 1254 was approximately 85% for 25 d, and the biodegradation rate of the dioxin-like PCBs ranged from 40% to 68%. Considering the limited information on PCB degradation by cyanobacteria, this study provides new knowledge for the remediation of PCB-contaminated soils. The key enzymes in degradation, the degradation pathway, and the engineering application effect of *Anabaena* PD-1 require in-depth study.

## Supporting Information

S1 File The analysis method of dioxin-like PCBs. (DOC)

S2 File The detailed 16S rRNA sequencing results of *Anabaena* PD-1. (DOC)

S1 Table Congeners of dioxin-like PCBs determined by GC/MS with characteristic mass fragments and retention time and recoveries. (DOC)
