# Supplementary material for: Biodegradation of polychlorinated biphenyls (PCBs) by the novel identified cyanobacterium Anabaena PD-1
Source: PLoS One. 2015 Jul 15;10(7):e0131450. doi: 10.1371/journal.pone.0131450 (PMC4503305; doi:10.1371/journal.pone.0131450)
Supplement: S1 File — (DOC) [file pone.0131450.s001.doc]

**S1 File**. The analysis method of dioxin-like PCBs. Quantification of dioxin-like PCBs was accomplished by use of previously established methods [38]. GC/MS analyses were performed on an Agilent Technologies 7890 gas-chromatograph coupled with a 5973 mass spectrometer using a DB5MS column (60 m × 0.25 mm ID × 0.25 μm film, Agilent Technologies, Palo Alto, CA, USA). Helium was used as carrier gas with a total flow of 24 mL/min. The injector temperature was 280 °C and the injection mode was splitless. Oven temperature increased from 80 °C to 280 °C, at 20 °C/min, after an initial hold at 80 °C for 3 min. Final temperature was maintained for 10 min. The detector temperature was 280 °C and the acquisition mode was SIM, monitoring the characteristic mass fragments of different dioxin-like PCBs listed of Table S1. Each congener was identified by the characteristic mass fragments (*m/z*) and the retention time (min). For each sample 1 μL was injected, using a solvent delay of 3 min.

determined by GC/MS with characteristic mass fragments and retention time and recoveries.

|  |  |  |  |
| --- | --- | --- | --- |
|  |  |  |  |
|  |  |  |  |
|  |  |  |  |
|  |  |  |  |
|  |  |  |  |
|  |  |  |  |
|  |  |  |  |
|  |  |  |  |
|  |  |  |  |
|  |  |  |  |
|  |  |  |  |
|  |  |  |  |
