# Supplementary material for: Biodegradation of polychlorinated biphenyls (PCBs) by the novel identified cyanobacterium Anabaena PD-1
Source: PLoS One. 2015 Jul 15;10(7):e0131450. doi: 10.1371/journal.pone.0131450 (PMC4503305; doi:10.1371/journal.pone.0131450)
Supplement: S2 File — (DOC) [file pone.0131450.s002.doc]

**S2 File. The detailed 16S rRNA sequencing results of *Anabaena* PD-1**.

5’-TGTGAGAATCTAGCTTCAGGTCGGGGACAACCACTGGAAACGGTGGCTAATACCGGATGTGCCGAAAGGTGAAAGATTTATTGCCTGAAGATGAGCTCGCGTCTGATTAGCTAGTTGGTGTGGTAAGAGCGCACCAAGGCGACGATCAGTAGCTGGTCTGAGAGGATGATCAGCCACACTGGGACTGAGACACGGCCCACACTCCTACGGGAGGCAGCAGTGGGGAATTTTCCGCAATGGGCGAAAGCCTGACGGAGCAATACCGCGTGAGGGAGGAAGGCTCTTGGGTTGTAAACCTCTTTTCTCAGGGAATAAAAAAATGAAGGTACCTGAGGAATAAGCATCGGCTAACTCCGTGCCAGCAGCCGCGGTAATACGGAGGATGCAAGCGTTATCCGGAATGATTGGGCGTAAAGCGTCCGCAGGTGGCACTGTAAGTCTGCTGTTAAAGAGCAAGGCTCAACCTGTAAAGGCAGTGGAAACTACAGAGCTAGAGTACGTTCGGGGCAGAGGGAATTCCTGGTGTAGCGGTGAAATGCGTAGAGATCAGGAAGAACACCGGTGGCGAAAGCGCTCTGCTAGGCCGTAACTGACACTGAGGGACGAAAGCTAGGGGAGCGAATGGGATTAGATACCCCAGTAGTCCTAGCCGTAAACGATGGATACTAGGCGTGGCTTGTATCGACCCGAGCCGTGCCGGAGCCAACGCGTTAAGTATCCCGCCTGGGGAGTACGCACGCAAGTGTGAAACTCAAAGGAATTGACGGGGGCCCGCACAAGCGGTGGAGTATGTGGTTTAATTCGATGCAACGCGAAGAACCTTACCAAGACTTGACATGTCGCGAATCTTCTTGAAAGGGAAGAGTGCCTTAGGGAGCGCGAACACAGGTGGTGCATGGCTGTCGTCAGCTCGTGTCGTGAGATGTTGGGTTAAGTCCCGCAACGAGCGCAACCCTCGTTTTTAGTTGCCAGCATTAAGTTGGGCACTCTAGAGAGACTGCCGGTGACAAATCGGAGGAAGGTGGGGATGACGTCAAGTCAGCATGCCCCTTACGTCTTGGGCTACACACGTACTACAATGCTACGGACAGAGGGCAGCAAGCTAGCGATAGCAAGCAAATCTCGTAAACCGTAGCTCAGTTCAGATCGCAGGCTGCAACTCGCCTGCGTGAAGGAGGAATCGCTAGTAATTGCAGGTCAGCATACTGCAGTGAATTCGTTCCCGGGCCTTGTACACACCGCCCGTCACACCATGGAAGCTGGCAACGCCCGAAGTCATTACTCCAACTTTTAGGAGAGGAGGATGCCTAAGGCAGTGCTGGTGACTGGGGTGAAGTCGTAACAAGGTAGCCGTACCGGAAGGTGTGGCTGGATCACCTCCTTTCT -3’
