# Supplementary material for: Biodegradation of polychlorinated biphenyls (PCBs) by the novel identified cyanobacterium Anabaena PD-1
Source: PLoS One. 2015 Jul 15;10(7):e0131450. doi: 10.1371/journal.pone.0131450 (PMC4503305; doi:10.1371/journal.pone.0131450)
Supplement: S1 Table — (DOC) [file pone.0131450.s003.doc]

**S1Table**. Congeners of dioxin-like PCBs determined by GC/MS with characteristic mass fragments and retention time and recoveries.

| PCB name | Congener | Characteristic mass fragments (*m/z*) | Recoveries  (%) |
| --- | --- | --- | --- |
| 3,3’,4,4’-Tetrachlorobiphenyl | 77 | 290,292,294 | 96% |
| 3,4,4’,5-Tetrachlorobiphenyl | 81 | 290,292,294 | 82% |
| 2,3,3’,4,4’-Pentachlorobiphenyl | 105 | 324, 326, 328 | 89% |
| 2,3,4,4’,5-Pentachlorobiphenyl | 114 | 324, 326, 328 | 85% |
| 2,3’,4,4’,5-Pentachlorobiphenyl | 118 | 324, 326, 328 | 93% |
| 2,3’,4,4’,5’-Pentachlorobiphenyl | 123 | 324, 326, 328 | 101% |
| 3,3’,4,4’,5-Pentachlorobiphenyl | 126 | 324, 326, 328 | 86% |
| 2,3,3’,4,4’,5-Hexachlorobiphenyl | 156 | 358,360,362,264 | 79% |
| 2,3,3’,4,4’,5’-Hexachlorobiphenyl | 157 | 358,360,362,264 | 95% |
| 2,3’,4,4’,5,5’-Hexachlorobiphenyl | 167 | 358,360,362,264 | 89% |
| 3,3’,4,4’,5,5’-Hexachlorobiphenyl | 169 | 358,360,362,264 | 77% |
| 2,3,3’,4,4’,5,5’-Heptachlorobiphenyl | 189 | 358,360,362,264 | 91% |
